# Effects of Acacetin on the Pharmacokinetics of Diazepam in vivo and in vitro

**DOI:** 10.5812/ijpr-164825

**Published:** 2025-11-18

**Authors:** Ailian Hua, Li Wang, Yao Zhu, Quan Zhou, Abdullah Al Mamun, Shuanghu Wang, Fan Wu, Minzhi Xu

**Affiliations:** 1 Department of Pharmacy, First People's Hospital of Linping District, Hangzhou, China; 2 Department of Pharmacy, Women’s Hospital, Zhejiang University School of Medicine, Hangzhou, China; 3 Central Laboratory of the Lishui Hospital of Wenzhou Medical University, The First Affiliated Hospital of Lishui University, Lishui People's Hospital, Lishui, China; 4 Department of Pharmacy, The First Affiliated Hospital of Zhejiang Chinese Medical University, Zhejiang Provincial Hospital of Chinese Medicine, Hangzhou, China; 5 Department of Pediatrics, The Lishui Hospital of Wenzhou Medical University, The First Affiliated Hospital of Lishui University, Lishui People's Hospital, Lishui, China

**Keywords:** Acacetin, Diazepam, Pharmacokinetics, Herb-Drug Interactions, Cytochrome P-450 Enzyme System

## Abstract

**Background:**

Herb-drug interactions (HDIs) have garnered significant attention in recent years.

**Objectives:**

To investigate the effects of acacetin on the pharmacokinetics of diazepam both in vivo and in vitro.

**Methods:**

Rat liver microsomes (RLMs) were incubated with diazepam and acacetin to determine the half-maximal inhibitory concentration (IC_50_) and inhibition constant (Ki) values of acacetin, as well as to evaluate its inhibitory effect on diazepam metabolism in vitro. For the in vivo experiment, twelve male Sprague-Dawley rats were randomly allocated into two groups (n = 6) and received either 50 mg/kg acacetin or vehicle for two weeks. Subsequently, diazepam (10 mg/kg) was administered to each rat. Blood samples (300 μL) were collected from the tail vein at 0.083, 0.25, 0.5, 1, 2, 3, 4, 6, and 8 hours post-administration. The plasma concentrations of diazepam and its metabolites were quantified using ultra-performance liquid chromatography-tandem mass spectrometry (UPLC-MS/MS).

**Results:**

The IC_50_ values for temazepam and nordiazepam in RLMs were 2.065 μM and 5.2 μM, respectively. The Ki values for temazepam and nordazepam demonstrated that acacetin inhibits diazepam metabolism in vitro. In vivo, pretreatment with acacetin increased the area under the curve (AUC) and maximum plasma concentration (Cmax) of diazepam, while significantly decreasing its apparent clearance (CLz/F, P < 0.05). The AUC values for temazepam and nordiazepam decreased, whereas their CLz/F values increased significantly (P < 0.05). PyMOL simulations indicated that acacetin and diazepam share the same cytochrome P450 3A4 (CYP3A4) or cytochrome P450 2C19 (CYP2C19) binding pocket, suggesting that acacetin inhibits diazepam metabolism via competitive inhibition.

**Conclusions:**

Acacetin significantly altered the pharmacokinetics of diazepam both in vivo and in vitro, indicating a potential interaction between acacetin and diazepam. Therefore, the concomitant use of acacetin and diazepam in clinical practice should be approached with caution.

## 1. Background

**Figure 1.**
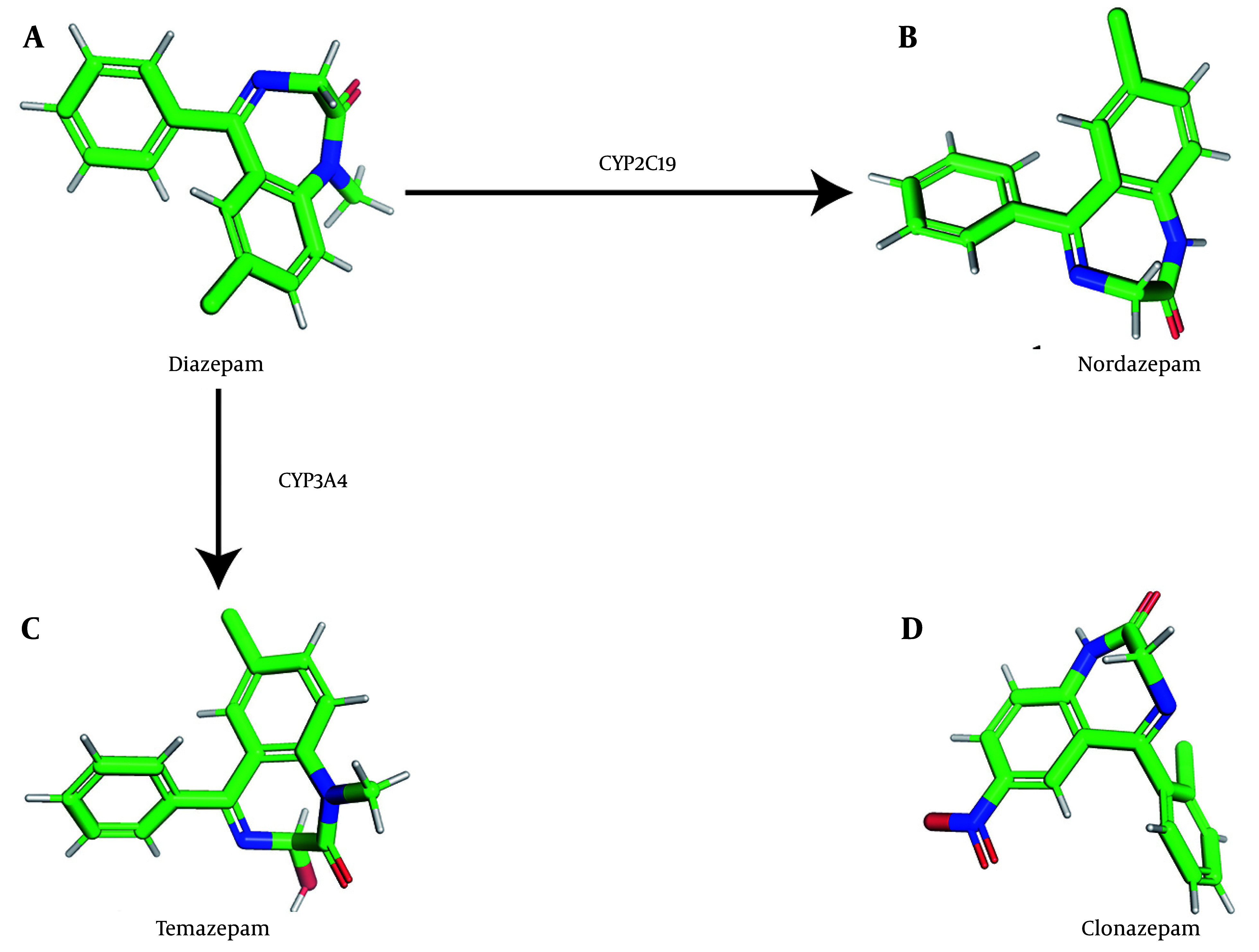
Structure diagrams of diazepam and its metabolites: Three-dimensional model of the chemical structure of diazepam (A); three-dimensional model of the chemical structure of nordazepam (B); three-dimensional model of the chemical structure of temazepam (C); and three-dimensional model of the chemical structure of clonazepam (D).

Diazepam, a benzodiazepine with diverse therapeutic applications, is widely used for the management of disorders such as insomnia, muscle spasms, anxiety, and seizures. Diazepam is primarily metabolized by cytochrome P450 2C19 (CYP2C19) or cytochrome P450 3A4 (CYP3A4) to form nordiazepam or temazepam, which are subsequently converted to oxazepam via hydroxylation or demethylation (1, 2). Additional P450 enzymes, including CYP1A2, CYP2B6, CYP2C8, and CYP2E1, also contribute to diazepam metabolism (3). For reference, we generated three-dimensional representations of the chemical structures of diazepam and its two major metabolites (Figure 1). 

The CYP is the most crucial enzyme system for drug metabolism in humans, and the majority of clinically used medications are metabolized by this system (4, 5). CYP450 enzymes include CYP3A4, CYP2D6, CYP2C9, CYP1A2, CYP2E1, CYP2C19, and additional subtypes. Notably, CYP3A4, CYP2D6, and CYP2C9 account for the majority of drug metabolism activity in the human liver (6, 7). Consequently, any compound that inhibits CYP enzyme activity is likely to affect the metabolism of substrate drugs.

When diazepam is coadministered with central nervous system depressants, such as monoamine oxidase type A inhibitors or tricyclic antidepressants, these agents can have mutually reinforcing effects. Combining antihypertensive drugs or diuretic antihypertensive drugs with diazepam enhances their antihypertensive effect. Furthermore, diazepam may interact more complexly with drugs that influence CYP450 enzymes; for example, hepatic enzyme inducers such as phenobarbital, phenytoin, and rifampicin can accelerate the elimination of diazepam, thereby reducing its plasma concentration (8, 9). Conversely, coadministration with inhibitors of hepatic drug-metabolizing enzymes can increase diazepam plasma concentration and prolong its half-life.

Herb-drug interactions (HDIs) have become an increasing concern in recent years. The HDIs are among the most significant clinical challenges that arise when prescription medicines and herbal products are taken concurrently. The widespread use of polypharmacy in treating most diseases further elevates the risk of HDIs in patients. The pharmacokinetic and pharmacological mechanisms underlying drug-drug interactions also underlie HDIs (10).

Acacetin is a naturally occurring flavonoid present in significant amounts in food sources and in the flowers of plants such as chrysanthemum and safflower (11). It can be extracted from wattle, acacia tree, thistle, airplane grass, *Buddleja officinalis*, and chrysanthemum, and is utilized in both medical research and the cosmetics industry. In addition to its traditional uses for alleviating depression, calming the mind, regulating qi, and dredging collaterals, acacetin exhibits a wide range of pharmacological activities, including anti-inflammatory, antimicrobial, antioxidant, and antimalarial effects, making it commonly used for the treatment of bone loss, cardiovascular disease, and various other conditions (12-15).

Previous studies have demonstrated that acacetin exerts anticancer effects by inhibiting receptors and transcription factors, modulating carcinogenic metabolism, promoting cancer cell proliferation, and regulating signaling pathways (16-20). Acacetin also inhibits glutamate release, regulates apoptosis in human T-cell leukemia Jurkat cells, and protects against kainic acid-induced neurotoxicity (21). The broad pharmacological profile of acacetin, particularly its antitumor activity, may render it a promising anticancer agent.

Like many xenobiotics, acacetin undergoes metabolism in vivo and can modulate CYP enzyme activity, acting as a potent inhibitor of the CYP1 family. Research indicates that it may also inhibit other CYP450 isoforms, such as CYP2B1, CYP2C9, CYP2C11, CYP2D1, CYP2E1, and CYP3A2. Additionally, acacetin has demonstrated both irreversible and reversible inhibition of CYP3A4. Recent studies suggest that acacetin suppresses CYP1A2 and CYP3A2, while inhibiting CYP2B1, CYP2C11, and CYP2E1 (22, 23). Despite the in vitro evidence of acacetin’s inhibitory activity against CYP450 enzymes, its potential interaction with diazepam — a drug primarily metabolized by CYP2C9 — has not been fully characterized in in vivo pharmacokinetic studies.

## 2. Objectives

In the present study, we aimed to investigate the effect of acacetin on the pharmacokinetics of diazepam both in vivo and in vitro. This research may prove pivotal in evaluating potential HDIs between acacetin and diazepam.

## 3. Methods

### 3.1. Drugs, Chemicals, and Reagents

Diazepam was obtained from Tianjin KingYork Pharmaceutical Co., Ltd. Nordazepam, temazepam, and clonazepam (internal standard, IS, purity > 98%) were purchased from Sigma-Aldrich (St. Louis, MO, USA). Analytical grade formic acid was also purchased from Sigma-Aldrich. Acacetin was supplied by Chengdu Must Technology Co., Ltd. (Chengdu, China). Nicotinamide adenine dinucleotide phosphate (NADPH) was acquired from Roche (Shanghai, China). Ultrapure water was prepared using a Milli-Q filtration system (Millipore, Bedford, MA, USA). UPLC-grade methanol and acetonitrile were obtained from Merck Company (Darmstadt, Germany). All other chemicals used were of analytical reagent grade.

### 3.2. Animals

Twelve healthy male Sprague-Dawley rats (220 ± 20 g) were provided by the Experimental Animal Center of Wenzhou Medical University, China. The animals were housed under controlled conditions with a 12-hour light/dark cycle, temperature maintained at 20 - 26°C, and relative humidity of 55 ± 15%. Rats received a standard rodent diet and had free access to tap water, except during the 12-hour fasting period prior to pharmacokinetic testing. All experimental protocols were reviewed and approved by the Lishui University Animal Care and Use Committee (ID: 2023YD0063), in accordance with the Guide for the Care and Use of Laboratory Animals. The study conformed to internationally accepted standards for animal research, adhering to the 3Rs principle. The ARRIVE guidelines were followed to ensure ethical reporting of experiments involving live animals.

### 3.3. Kinetic Studies and Enzyme Inhibition of Acacetin in vitro

The procedures described by Zhou et al. (24) were employed for the preparation and quantification of rat liver microsome (RLM) protein concentrations. The incubation solution comprised diazepam, 100 mM potassium phosphate buffer (pH 7.4), 0.3 mg/mL RLMs, acacetin (inhibitor), and 1 mM NADPH. Inhibitory concentration (IC_50_) was determined using substrate concentrations approximating their respective Km values (128.8 μM and 65.72 μM for diazepam conversion to temazepam and nordiazepam, respectively). Acacetin was tested at concentrations of 0.01, 0.1, 1, 2, 10, 20, 50, and 100 μM.

To determine the inhibition constant (Ki), acacetin (0, 1, 2, and 4 μM) was incubated with varying concentrations of diazepam (12.5, 25, 50, and 100 μM) for assessment of temazepam formation, and acacetin (0, 2, 5, and 10 μM) was incubated similarly for nordiazepam formation. The mixtures were preincubated at 37°C for 5 minutes. The reaction was initiated by adding NADPH for a 30-minute incubation, then terminated by transferring to ice and adding 200 μL of acetonitrile. Clonazepam (20 μL) was added as the internal standard, the mixture was vortexed for two minutes, and centrifuged at 13,000 rpm for 5 minutes. The ultra-performance liquid chromatography-tandem mass spectrometry (UPLC-MS/MS) analysis was performed using a 2 μL aliquot of the supernatant.

### 3.4. In vivo Pharmacokinetic Experiments

Following approval by the Animal Care and Use Committee of Wenzhou Medical University, the twelve rats were divided into two groups of six. The experimental group received daily intraperitoneal injections of 50 mg/kg acacetin for two weeks, while the control group received vehicle (a mixture of dimethyl sulfoxide, polyethylene glycol 200, and ultrapure water) as pretreatment. All rats were then administered diazepam by oral gavage at a dose of 10 mg/kg, 30 minutes after the final pretreatment. Blood samples (300 μL) were collected from the tail vein at 0.083, 0.25, 0.5, 1, 2, 3, 4, 6, and 8 hours post-administration. Plasma was separated by centrifugation at 3000 rpm for 10 minutes and immediately stored at -80°C. For analysis, plasma samples were thawed at room temperature and mixed with 200 μL acetonitrile and 20 μL internal standard, followed by 30 seconds of vortexing and centrifugation at 13,000 rpm for 5 minutes. UPLC-MS/MS analysis was conducted on 2 μL of the supernatant.

### 3.5. Instrumentation and Ultra-Performance Liquid Chromatography-Tandem Mass Spectrometry Conditions

Separation of diazepam and its metabolites was achieved using an ACQUITY UPLC BEH C18 column (50 mm × 2.1 mm, 1.7 μm). The mobile phase consisted of a gradient of (A) acetonitrile and (B) 0.1% formic acid, delivered at a flow rate of 0.4 mL/min. The elution gradient was as follows: 0 - 0.3 min, 10% → 30% A; 0.3 - 2 min, 30% → 95% A; 2 - 2.5 min, 95% A; and 2.5 - 2.6 min, 95% → 10% A. The column was maintained at 40°C for 3 minutes. Clonazepam (500 ng/mL) was used as the internal standard.

Mass spectrometry was performed in positive ion mode using a triple quadrupole mass spectrometer with an electrospray ionization interface. Quantitative analysis was conducted in multiple reaction monitoring mode. The nitrogen cone gas flow rates were 50 L/h and 1000 L/h. The ion source and desolvation gas temperatures were 150°C and 500°C, respectively, and the capillary voltage was 2.5 kV. The monitored transitions were: 285.1 - 193.1 m/z for diazepam, 271.1 - 139.9 m/z for nordazepam, 301.1 - 254.9 m/z for temazepam, and 316.1 - 269.9 m/z for clonazepam. Data were processed using MassLynx version 4.1 software (Waters Corp., Milford, MA, USA). The analytical method validation was performed as described by Zhou et al. (24).

### 3.6. Molecular Docking Method

Molecular docking was performed as described in previous literature (25) to identify potential inhibitory mechanisms. The molecular structures of diazepam, nordazepam, and temazepam were retrieved from PubChem (NCBI), and the crystal structures of CYP2C19 (PDB ID: 4gqs) and CYP3A4 (PDB ID: 2j0d) were obtained from the RCSB Protein Data Bank. Docking was conducted using AutoDock Vina Version 1.2.3 (Scripps Research Institute, USA), with a grid box of 60 Å × 60 Å × 60 Å and spacing of 0.3753 Å. Structure optimization and visualization were performed with PyMOL open-source Version 2.5.2 (Schrodinger, USA). The docked structure with the highest affinity was selected for further analysis.

### 3.7. Data Analysis

Pharmacokinetic assessment was performed using noncompartmental analysis with DAS software (version 3.2.8, China). Plasma concentration–time curves were generated using mean drug concentrations at each time point. The following pharmacokinetic parameters were examined: Plasma clearance (CLz/F), plasma half-life (t1/2), area under the curve (AUC) of the plasma concentration-time, maximum plasma concentration (Cmax), and time to maximum plasma concentration (Tmax). The IC_50_, Ki, and αKi values were calculated using GraphPad Prism 7.0 software (GraphPad Software Inc., San Diego, CA, USA). Statistical analyses of the main pharmacokinetic parameters were conducted using SPSS 24.0 software (IBM, Chicago, IL, USA) with Student’s *t*-test. Differences were considered statistically significant at P < 0.05.

## 4. Results

### 4.1. Effects of Acacetin on the Pharmacokinetics of Diazepam in vitro

**Figure 2.**
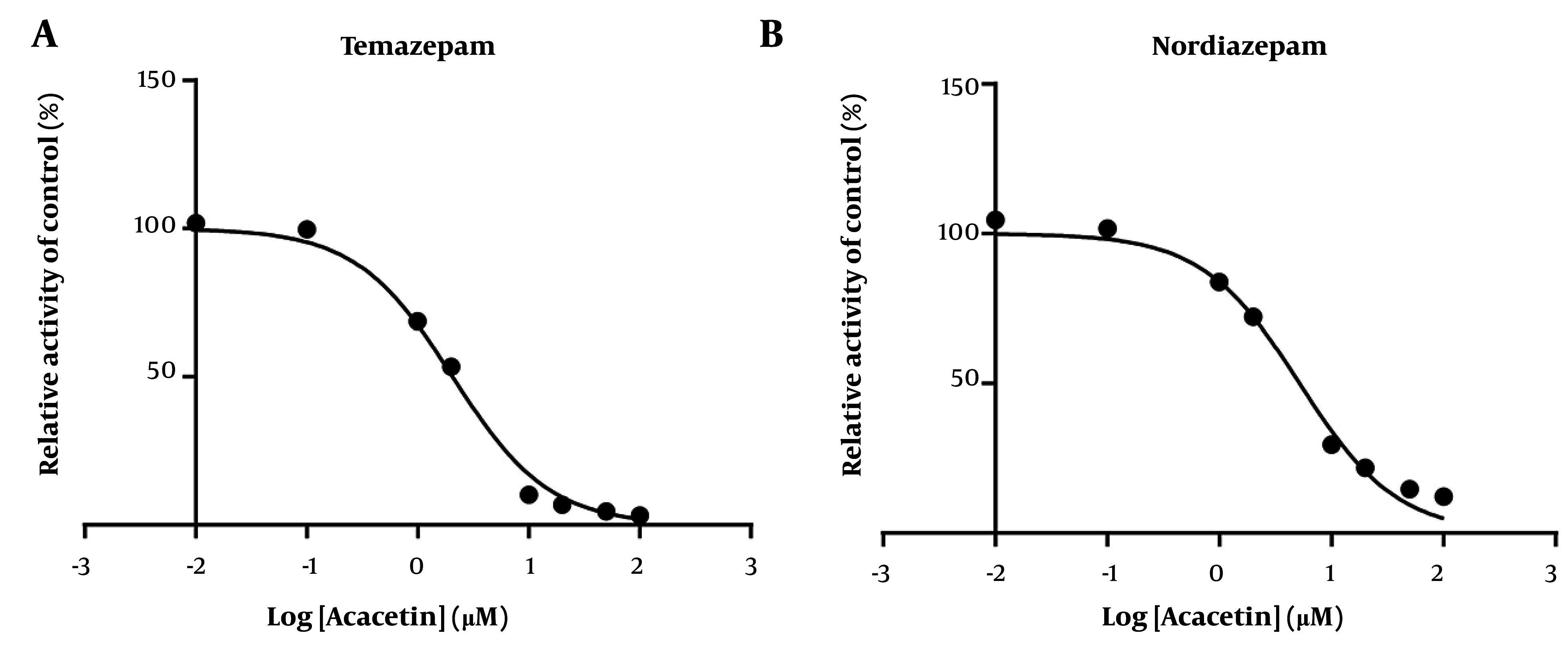
Acacetin at various concentrations was administered to determine the half-maximal inhibitory concentration (IC_50_) of diazepam with respect to temazepam (A) and nordiazepam (B) in rat liver microsomes (RLMs, mean ± standard deviation; n = 3).

**Figure 3.**
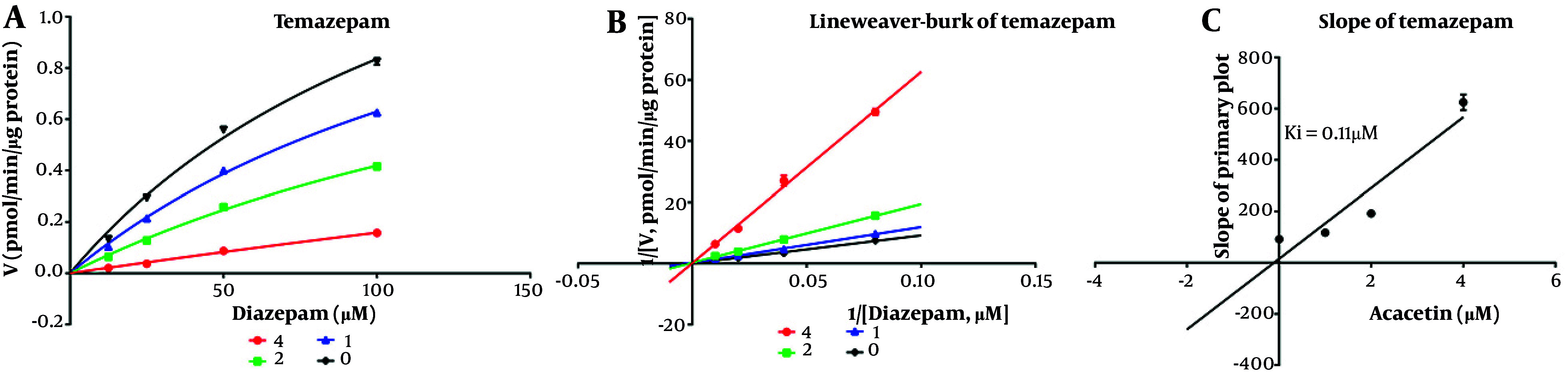
Michaelis-Menten model for temazepam (A), Lineweaver-Burk plot for temazepam (B), and the slope for temazepam for inhibition constant (Ki) (C) at various concentrations of acacetin in rat liver microsomes (RLMs)

**Figure 4.**
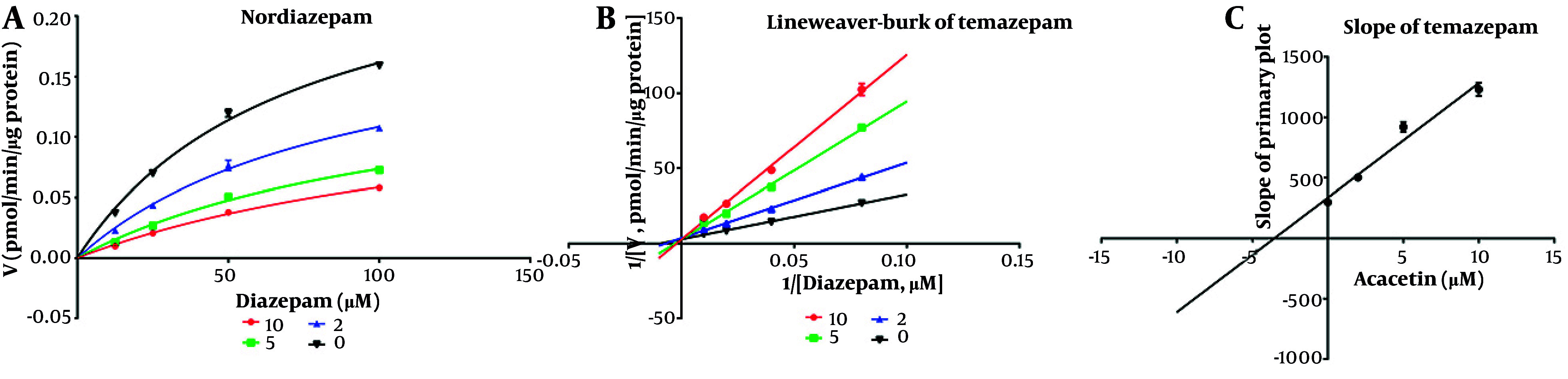
Michaelis-Menten model for nordiazepam (A), Lineweaver-Burk plot for nordiazepam (B), and the slope for nordiazepam for inhibition constant (Ki) (C) at various concentrations of acacetin in rat liver microsomes (RLMs)

As shown in Figure 2, the IC_50_ values for diazepam metabolism to temazepam and nordiazepam were determined using a range of acacetin concentrations (0.01 μM to 100 μM). Acacetin moderately inhibited CYP3A4 and CYP2C19, with IC_50_ values of 2.065 μM and 5.2 μM, respectively (Figure 2A and B). Enzyme kinetics and secondary plot analysis (Figures 3 and 4) revealed that acacetin competitively inhibits the metabolism of diazepam to temazepam and nordiazepam, with Ki values of 0.11 μM and 3.54 μM, respectively.

### 4.2. Effects of Acacetin on the Pharmacokinetics of Diazepam in vivo

**Figure 5.**
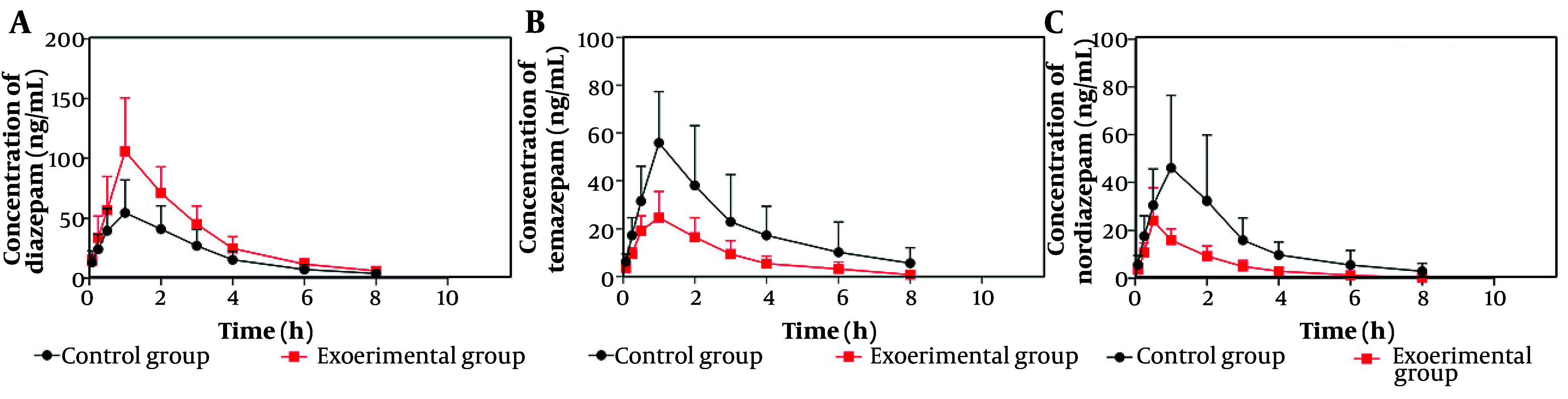
Mean plasma concentration-time curves for diazepam (A), temazepam (B), and nordiazepam (C) in the experimental group and control group after oral administration of diazepam (mean ± standard deviation, n = 6)

Figure 5 presents the mean plasma concentration-time curves for temazepam, nordiazepam, and diazepam in the control and experimental groups. As shown in Figure 5A, the experimental group exhibited higher mean plasma concentrations of diazepam compared to the control group. Figure 5B and C demonstrate that the experimental group had significantly lower mean plasma concentrations of temazepam and nordiazepam than the control group. The pharmacokinetic parameters are summarized in Tables 1 and 2. 

**Table 1.** Main Pharmacokinetic Parameters of Diazepam in the Control Group and Experimental Group (N = 6) ^a^

Parameters	Unit	Control Group	Experimental Group
**AUC (0 - t)**	ug/L × h	172.25 ± 62.45	293.14 ± 85.29 ^b^
**AUC (0 - ∞)**	ug/L × h	182.02 ± 58.15	309.00 ± 83.77 ^b^
**MRT (0 - t)**	h	2.51 ± 0.58	2.42 ± 0.26
**MRT (0 - ∞)**	h	3.11 ± 1.59	2.89 ± 0.54
**t1/2z**	h	1.81 ± 1.26	1.84 ± 0.64
**Tmax**	h	1.33 ± 0.52	1.17 ± 0.41
**Vz/F**	L/kg	164.39 ± 141.97	91.91 ± 38.08
**CLz/F**	L/h/kg	59.78 ± 18.43	34.31 ± 8.80 ^b^
**Cmax**	ug/L	57.75 ± 24.81	111.15 ± 42.02 ^b^

Abbreviations: AUC, area under the curve; Tmax, time to maximum plasma concentration; Cmax, maximum plasma concentration.

^a^ Values are expressed as mean ± SD.

^b^ Significantly different from the control, P < 0.05.

**Table 2.** Main Pharmacokinetic Parameters of Temazepam and Nordiazepam in the Control Group and Experimental Group (N = 6) ^a^

Parameters	Unit	Temazepam		Nordiazepam	
		Control Group	Experimental Group	Control Group	Experimental Group
**AUC (0 - t)**	ug/L × h	171.31 ± 80.86	69.62 ± 29.23 ^b^	127.95 ± 61.83	45.90 ± 15.77 ^b^
**AUC (0 - ∞)**	ug/L × h	191.26 ± 103.22	71.84 ± 29.77 ^b^	139.73 ± 64.72	46.78 ± 16.15 ^b^
**MRT (0 - t)**	h	2.56 ± 0.70	2.24 ± 0.36	2.42 ± 0.77	1.93 ± 0.24
**MRT (0 - ∞)**	h	3.31 ± 1.44	2.51 ± 0.34	3.22 ± 1.87	2.08 ± 0.32
**t1/2z**	h	2.11 ± 0.82	1.62 ± 0.27	1.97 ± 1.37	1.34 ± 0.31
**Tmax**	h	1.50 ± 0.84	0.75 ± 0.27	1.25 ± 0.88	0.58 ± 0.20
**Vz/F**	L/kg	193.13 ± 111.15	409.02 ± 271.30	238.18 ± 204.62	456.59 ± 190.36
**CLz/F**	L/h/kg	67.07 ± 38.29	167.23 ± 89.04 ^b^	85.13 ± 36.98	235.89 ± 77.84 ^b^
**Cmax**	ug/L	60.51 ± 20.77	26.78 ± 9.53 ^b^	47.75 ± 28.99	24.85 ± 13.22

Abbreviations: AUC, area under the curve; Tmax, time to maximum plasma concentration; Cmax, maximum plasma concentration.

^a^ Values are expressed as mean ± SD.

^b^ Significantly different from the control, P < 0.05.

As indicated in Table 1, the AUC (0 - t) and AUC (0 - ∞) for diazepam increased by 70.18% and 69.76%, respectively, relative to the control group. The Cmax increased by 92.47%, while the CLz/F decreased by 42.61%. Table 2 shows that the AUC (0 - t) and AUC (0 - ∞) for temazepam decreased by 59.36% and 62.44%, respectively, and the CLz/F increased by 149.34% with a 55.74% decrease in Cmax compared to the control group. For nordiazepam, the AUC (0 - t) and AUC (0 - ∞) decreased by 64.13% and 66.52%, respectively, and the CLz/F increased by 177.09%. There was no statistically significant difference in the Cmax between groups for nordiazepam.

### 4.3. Molecular Docking Prediction of Diazepam and Its Metabolites

**Figure 6.**
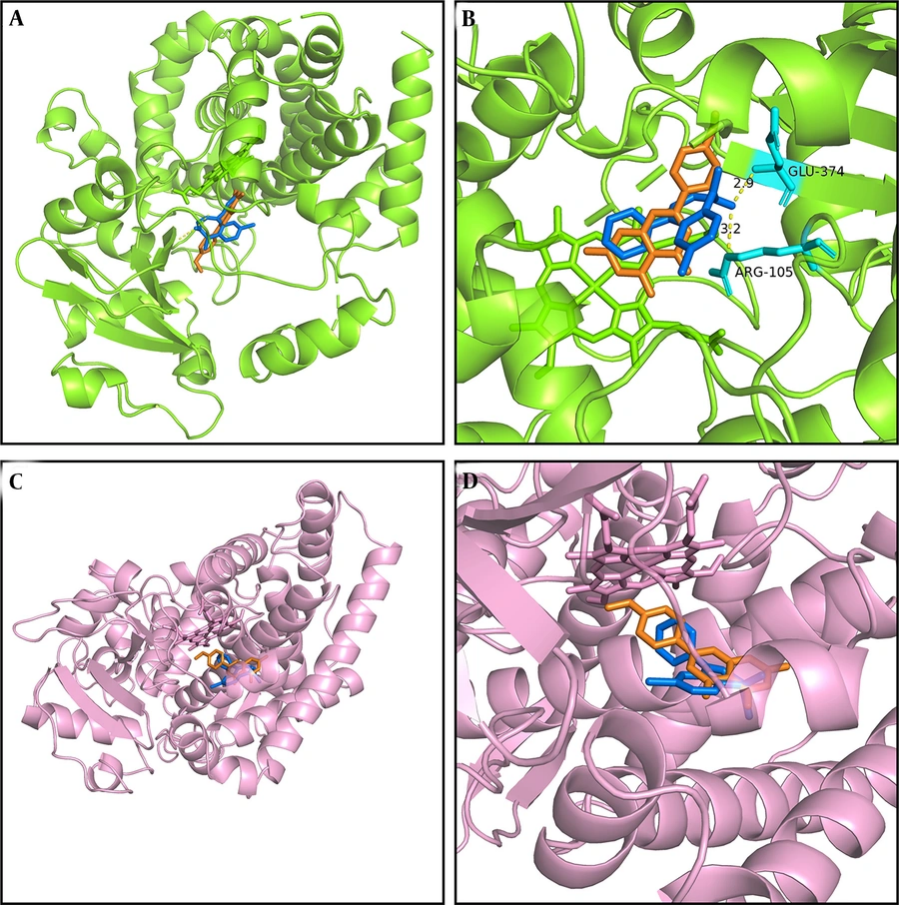
Molecular docking scheme for diazepam and acacetin: Action site between acacetin and cytochrome P450 3A4 (CYP3A4), formed via hydrogen bonding (A); enlarged region of A (B); action site between acacetin and cytochrome P450 2C19 (CYP2C19) (C); and enlarged region of C (D). Blue represents diazepam, and orange represents acacetin.

We conducted molecular docking analysis in this study using previously reported methods (2, 27) to elucidate the mechanism underlying the interaction between acacetin and diazepam, which involves hydrogen bonding, pi–pi interactions, pi–cation bonding, and pi–sigma interactions, each characterized by varying binding energies (Figure 6). As illustrated in Figure 6A and B, acacetin interacts with glutamic acid (GLU) at position 374 and arginine (ARG) at position 374 of CYP3A4 via hydrogen bonding, with the respective interaction sites being 2.9 Å and 3.2 Å apart according to the simulation results obtained using PyMOL. As shown in Figure 6A and B, both acacetin and diazepam bind closely to CYP3A4 within the same binding pocket, exhibiting binding energies of -7.3 kcal/mol and -8.5 kcal/mol, respectively. The interaction sites of the CYP2C19 enzyme with acacetin and diazepam are depicted in Figure 6C and D, with binding energies of -8.5 kcal/mol and -7.6 kcal/mol, respectively. Furthermore, acacetin and diazepam also bind closely to CYP2C19 within the same binding pocket, supporting the conclusion that acacetin inhibits the metabolism of diazepam through competitive inhibition.

## 5. Discussion

In recent years, interest in herbal remedies as complementary or alternative medicines has grown, leading to increased use of herbal products alongside conventional pharmaceuticals (10, 28). This trend elevates the risk of HDIs, particularly involving CYP enzymes.

Acacetin, a naturally occurring flavone, possesses multiple pharmacological effects. Previous studies have shown that acacetin inhibits glutamate release and protects against kainic acid-induced neurotoxicity in rats (29). Additionally, acacetin has demonstrated chemopreventive effects in cancer models by inhibiting glutamate release and preventing neurotoxicity. Acacetin has also been reported to inhibit the metabolism of drugs such as flurbiprofen and testosterone, which are CYP2C9 and CYP3A4 substrates, respectively (30). However, limited research has focused on HDIs between diazepam and conventional herbal medicines.

This study is the first to examine the effects of acacetin on diazepam both in vitro and in vivo. In vitro, acacetin significantly inhibited diazepam metabolism in RLMs, with IC_50_ values of 2.065 μM and 5.2 μM for temazepam and nordazepam, respectively. The Ki values for temazepam and nordazepam were 0.11 μM and 3.54 μM, respectively, indicating competitive inhibition of diazepam metabolism by acacetin. Previous research (23) found that acacetin competitively inhibits CYP2B1, CYP2C11, and CYP2E1, and exhibits mixed inhibition of CYP1A2 and CYP3A2. These results suggest that acacetin and diazepam bind to the same active site on the enzyme, with overlapping binding positions (Figure 6). 

The competitive inhibition of CYP3A4 and CYP2C19 by acacetin suggests that acacetin may interact with other CYP3A4 or CYP2C19 substrates, including tyrosine kinase inhibitors such as osimertinib, apatinib, and tofacitinib (31-33), as well as proton pump inhibitors such as omeprazole (34).

Diazepam is frequently administered orally for central nervous system disorders, such as anxiety, epilepsy, and alcohol withdrawal. Its long and variable half-life (20 - 50 hours) can result in prolonged sedation, ataxia, psychosis, hypotension, and decreased respiratory rate; its active metabolite, nordiazepam, has an even longer half-life (50 - 100 hours). Given these pharmacokinetic properties, interactions involving diazepam and other drugs or herbs require careful consideration.

When coadministered with acacetin, the AUC (0 - t), AUC (0 - ∞), and Cmax values for diazepam increased significantly, while CLz/F decreased by 42.61%. For temazepam and nordiazepam, acacetin decreased AUC (0 - t) and AUC (0 - ∞) but increased CLz/F. These findings from in vivo experiments align with the in vitro results, confirming that acacetin influences diazepam metabolism. Notably, the Cmax for temazepam decreased significantly, while the Cmax for nordiazepam did not differ significantly between groups. This suggests that acacetin more potently inhibits CYP3A4 than CYP2C19, consistent with the in vitro data. Acacetin likely alters diazepam pharmacokinetics by inhibiting CYP3A4 activity and reducing first-pass hepatic metabolism, thereby increasing diazepam bioavailability.

Other herbs, such as imperatorin, have also been shown to inhibit diazepam metabolism both in vivo and in vitro. This study is the first to investigate the effects of acacetin on diazepam pharmacokinetics, providing essential data for clinical practice. Nevertheless, some limitations remain. Due to resource constraints, this study could not be conducted in a clinical setting. Future research should further elucidate specific inhibitory mechanisms, and clinical trials are necessary to confirm these findings.

### 5.1. Conclusions

Given that CYP3A4 and CYP2C19 inhibitors are frequently prescribed in clinical practice, the effects of coadministered medications on diazepam disposition should be closely monitored. Acacetin significantly altered the pharmacokinetics of diazepam both in vivo and in vitro. The HDIs may occur when acacetin and diazepam are used together; therefore, clinicians should exercise caution with their concurrent administration.

## Data Availability

The dataset generated and analyzed during the current study is available from the corresponding author on reasonable request during submission or after publication. The data are not publicly available due to patient confidentiality.
